# CBD-enriched cannabis for autism spectrum disorder: an experience of a single center in Turkey and reviews of the literature

**DOI:** 10.1186/s42238-021-00108-7

**Published:** 2021-12-16

**Authors:** Serap Bilge, Barış Ekici

**Affiliations:** 1 Department of Pediatric Neurology, Çukurova Medical School, 01790 Balcalı, Turkey; 2 Department of Pediatric Neurology, Pediatric Neurology Clinics, Istanbul, Turkey

**Keywords:** Autism spectrum disorder, Cannabidiol, Cannabis

## Abstract

**Introduction:**

Autism spectrum disorder is a neurodevelopmental disorder characterized by deficits in communication, social interaction, restricted interest, and repetitive behaviors. Although more cases are being diagnosed, no drugs are approved to treat the core symptoms or cognitive and behavioral problems associated with autism. Therefore, there is an urgent need to develop an effective and safe treatment.

**Objective:**

In this study, we aim to share our 2-year experience with CBD-enriched cannabis treatment in autism and review the latest studies.

**Materials and methods:**

The study included 33 (27 males, six females) children diagnosed with autism spectrum disorder who were followed up between January 2018 and August 2020. The mean age was 7.7 ± 5.5 years. The average daily dosage of cannabidiol (CBD) was 0.7 mg/kg/day (0.3–2 mg/kg/day). The median duration of treatment was 6.5 months (3–28 months). The preparations used in this study contained full-spectrum CBD and trace elements tetrahydrocannabinol (THC) of less than 3%.

**Results:**

The outcomes were evaluated before and after treatment based on clinical interviews. At each follow-up visit, parents were asked to evaluate the effectiveness of the CBD-enriched cannabis treatment. According to the parents’ reports, no change in daily life activity was reported in 6 (19.35%) patients. The main improvements of the treatment were as follows: a decrease in behavioral problems was reported in 10 patients (32.2%), an increase in expressive language was reported in 7 patients (22.5%), improved cognition was reported in 4 patients (12,9%), an increase in social interaction was reported in 3 patients (9.6%), and a decrease in stereotypes was reported in 1 patient (3.2%). The parents reported improvement in cognition among patients who adhered to CBD-enriched cannabis treatment for over two years. The antipsychotic drug could be stopped only in one patient who showed mild ASD symptoms. No change could be made in other drug use and doses. Additionally, this study includes an extensive review of the literature regarding CBD treatment in autism spectrum disorder. According to recent studies, the average dose of CBD was 3.8±2.6 mg/kg/day. The ratio of CBD to THC in the used preparations was 20:1. The most significant improvements were seen in the behavioral problems reported in 20–70% of the patients.

**Conclusion:**

Using lower doses of CBD and trace THC seems to be promising in managing behavioral problems associated with autism. In addition, this treatment could be effective in managing the core symptoms and cognitive functions. No significant side effects were seen at the low doses of CBD-enriched cannabis when compared to other studies.

## Background

Autism spectrum disorder (ASD) is a neurodevelopmental disorder that varies in severity and is characterized by deficits in communication, social interaction, restricted interest, and repetitive behaviors (Fusar-Poli et al. 2020). During the last three decades, there has been a threefold increase in the number of children diagnosed with ASD (Lihi Bar-Lev Schleider et al. 2019). Currently, it affects up to 1 in 54 individuals (Maenner et al. 2020). Cooccurring medical conditions such as epilepsy, intellectual disability, and behavior problems occur in these individuals (Pretzsch et al. 2019a; Pretzsch et al. 2019b).

The etiopathogenesis of ASD remains largely unknown. Several genetic, perinatal, and environmental factors seem to be involved. Some researchers have evidenced an imbalance in the endogenous neurotransmission system, such as the serotoninergic, γ aminobutyric acid (GABA), and endocannabinoid system (ECS), which regulate functions such as emotional responses and social interactions typically impaired in ASD (Fusar-Poli et al. 2020).

Endocannabinoids (eCBs) and their receptors are present in the nervous system, connective tissue of internal organs, glands, and immune system. Cannabinoid receptor 1 (CB1) is a G protein-coupled receptor (GPR) that is found mainly in the central nervous system (Mc Partlan et al. 2014). In mammals, high concentrations of CB1 are found in the brain area that regulates appetite, memory, fear extinction, motor responses, and postures such as the hippocampus, basal ganglia, basolateral amygdala, hypothalamus, and cerebellum (Aran et al. 2019; Mc Partlan et al. 2014). CB1 can also be found in nonneuronal cells. Data indicate that cannabinoid receptor type 2 (CB2) is linked to a variety of immune functional events. However, it may play a functionally relevant role in the central nervous system (Aran et al. 2019; Bridgemanan and Abazia 2017).

There are two endogenous cannabinoids, N-arachidonoylethanolamine (anandamide) and two arachidonoylglycerols (2-AG). The ECS has been broadened by discovering new secondary receptors, ligands, and ligand metabolic enzymes, including transient receptor potential cation channel subfamily V member 1 (TRPV1) (Mc Partlan et al. 2014).

Anandamide and 2-AG can act via CB1 and CB2 receptors and exert a range of biological effects in central and peripheral cells. Anandamide is broken down by fatty acid amide hydrolase (FAAH); inhibitors of FAAH lead to an increase in anandamide. CBD act as an inhibitor of FAAH (Bridgemanan and Abazia 2017). Endocannabinoid signaling occurs in a retrograde direction; that is, signaling is initiated in postsynaptic neurons and acts upon presynaptic terminals. In contrast to classical neurotransmitters, eCBs are not stored. They are produced on demand upon stimulation of postsynaptic cells (Aran et al. 2019; Zamberletti et al. 2017).

**Fig. 1:**
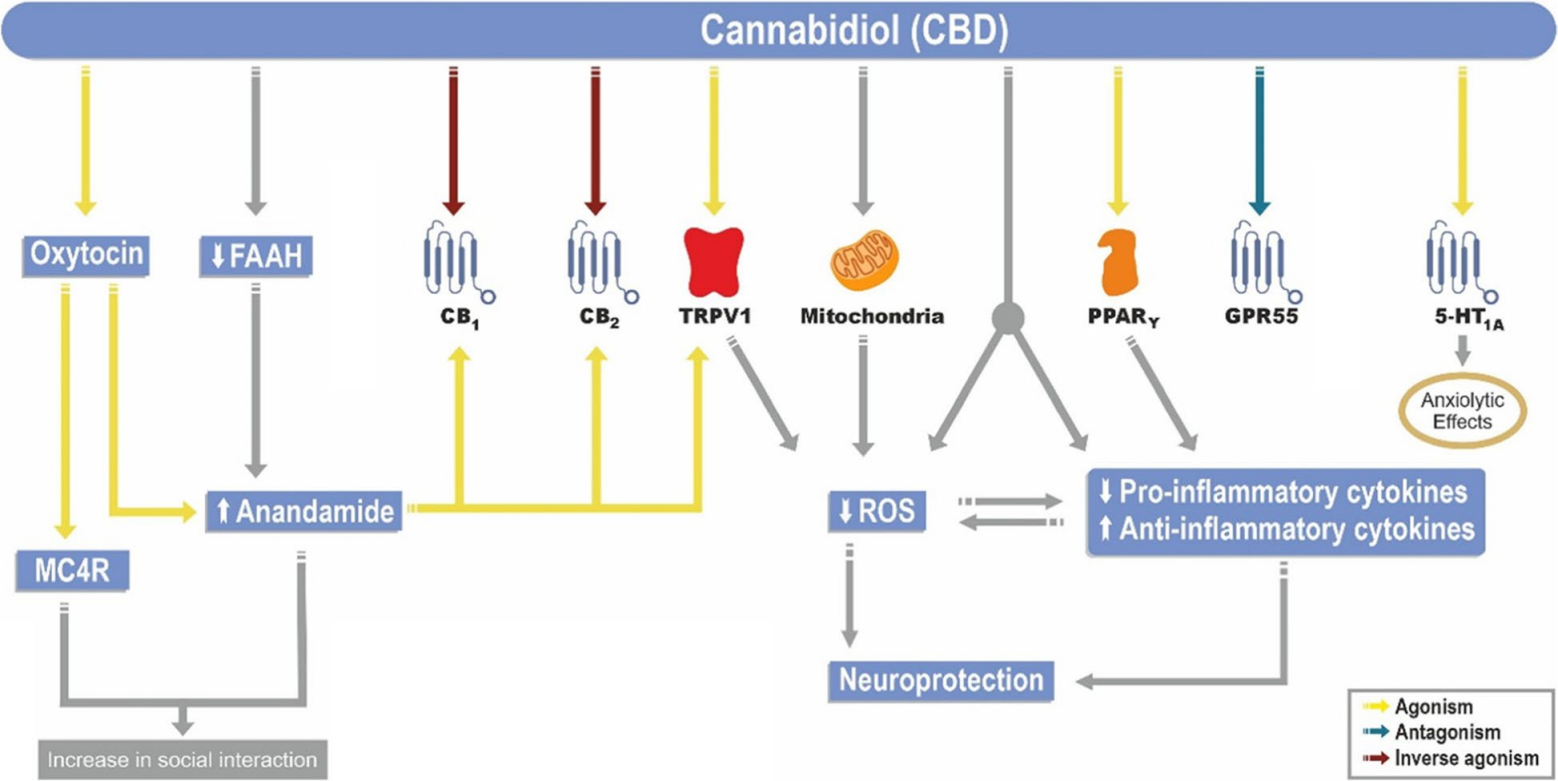
CBD and mechanism of action. CBD, cannabidiol; FAAH, fatty acid amide hydrolase CB, cannabinoid receptor; TRPV1, transient receptor potential cation channel subfamily V member 1; PPAR-γ, peroxisome proliferator-activated receptor-gamma; GPR, G protein-coupled receptor; GPR55, G protein-coupled receptor 55; 5-HT_1A_, serotonin 5HT receptor; MC4R, melanocortin 4 receptor; ROS, reactive oxygen species

Interestingly, CBD displays a low affinity for CB1 and CB2 receptors. CBD facilitates excitatory glutamate and inhibitory GABA neurotransmission across the brain through agonism at the TRPV1 receptor (Pretzsch et al. 2019a; Mc Partlan et al. 2014). Additionally, CBD can increase GABAergic transmission by antagonizing G protein-coupled receptor 55 (GPR55), especially in the basal ganglia. CBD is thought to be an agonist at prefrontal serotonin 5-HT_1A_ receptors (Castillo et al. 2012) (Fig. 1).

Another mechanism of action can be via vasopressin and oxytocin. The presence of oxytocin in the CSF seems to originate from neuronal oxytocinergic extensions to the limbic system, brain stem, and spinal cord. Oxytocin receptors are distributed in different parts of the central nervous system, such as the basal ganglia, limbic system, thalamus and hypothalamus, and brain stem. Oxytocin modulates social behavior, motor function, pain control, memory and learning, eating behavior, stress and anxiety, and emotional processing. Oxytocin administration reduces stress and anxiety and depression in animal models. This effect seems to be modulated at least partly by the effects of oxytocin on the hypothalamic-pituitary-adrenal (HPA) axis and the opioidergic and dopaminergic systems in limbic brain structures. Several animal model studies support the role of oxytocin in improving social behavior, an effect that appears to involve the melatoninergic and endocannabinoid systems, specifically an increase in social interactions produced by agonism at the melanocortin four receptor (MC4R (Russo et al. 2005; Dos Santos et al. 2019). CBD leads to enhancement in the release of vasopressin and oxytocin; thus, it could positively affect ASD core symptoms. Studies have shown that oxytocin administration to patients with ASD improves social interactions, reduces classic repetitive behavior, and increases eye contact (Weia et al. 2015). Another mechanism of action of CBD is to act as a dopamine receptor antagonist, which can facilitate its use as an antipsychotic (Dos Santos et al. 2019; Weia et al. 2015).

CBD may act as a neuroprotectant against mitochondrially acting toxins (Davies and Bhattacharyya 2019; Bartova and Birmingham 1976). The highly lipophilic aspect of CBD gives them access to intracellular sites of action. Many studies have suggested mitochondria as targets for CBD, and many theories are based on this idea; one of these theories is that the outer mitochondrial membrane has CB1 receptors. This theory reveals that CBD affects the function of the cells by establishing homeostasis and influencing mitochondria and energy production (Bartova and Birmingham 1976; Ryan et al. 2009).

THC is known to be a major psychoactive component of Cannabis. THC is a partial agonist at CB1 and CB2 (Ryan et al. 2009). Signals through transducing G-proteins and activation of these G-proteins by THC cause inhibition of adenyl cyclase activity, the closing of voltage-gated calcium channels, and the opening of inward rectifying potassium channels. The psychoactive nature of THC limits its use due to side effects. However, a varied mixture of THC with other phytocannabinoids with very weak or no psychoactivity quality has started to be used as a therapeutic drug in humans (Bloomfield et al. 1982; Rodríguez De Fonseca et al. 1992). In this study, we aim to share our 2-year experiences with CBD-enriched cannabis treatment in autism and review the latest studies.

## Methods and materials

### Patients

This research was conducted in accordance with the Declaration of Helsinki at the Pediatric Clinics of Neurology in Istanbul. CBD-enriched cannabis treatment was started in 54 patients who were diagnosed with ASD. The study included 33 (27 males, six females) children diagnosed with autism spectrum disorder who were followed up between January 2018 and August 2020. The diagnosis of ASD was based on DSM V criteria (American Psychiatric Association 2013). Twenty-one participants refused to participate in this study. The most common reasons for not participating in the study were fear of adverse effects, cost of CBD-enriched cannabis, bitter taste, and behavioral problems. The mean age of the non-participating 21 children was 7.2 ± 4.2. Ten patients had mild, while 11 had severe autism according to the DSM V. Four patients were female, and 17 were male. Three children had abnormal EEG, and one was diagnosed with epilepsy, and he was on valproic acid treatment. Three patients attended mainstream schools and received their education there, while eighteen patients had intellectual disabilities. All non-participating 21 ASD patients used antipsychotic drugs. Sixteen patients used risperidone, and five patients used aripiprazole. The median duration of antipsychotic drug administration was 8.2 ± 2.6 months. The median duration of follow-up was 4.4 1 ± 1 years.

Informed consent was obtained from the parents of all children participating in the study. The mean age of the participating 33 children was 7.7 ± 5.5. Fifteen patients had mild autism, while 18 had severe autism according to the DSM V. Three patients were diagnosed with epilepsy before starting CBD-enriched cannabis; two of them used oxcarbazepine, while one used valproic acid. Seven patients had abnormal electroencephalography (EEG) results without any episodes of previous seizures. Five patients attended mainstream schools and received their education there, while twenty-eight patients had intellectual disabilities and attended schools that catered to special educational needs. Two patients were using CBD-enriched cannabis for over two years. There was no predefined duration of this treatment in our patients. All ASD patients used antipsychotic drugs. Twenty-six patients used risperidone, and seven patients used aripiprazole. The median duration of antipsychotic drug administration was 8.5 ± 2.3 months. All the patients were provided with psychosocial treatment. The median duration of follow-up was 4.6 ± 1.3 years. There were no significant differences between the 2 group profiles (participating and non-participating) regarding sex ratio, median age, and autism severity.

### Treatment

The legal basis for using cannabis-related drugs is not fully apparent in Turkey, and a maximum of 0.3% THC is allowed to be used in these preparations. Due to the lack of availability and difficulty of access to these therapeutic preparations, various cannabis strains of CBD-enriched cannabis extracts have been used. The two CBD-enriched cannabis brands used were CBDistillery and CBDodgamax. Both had similar available forms of drops of 500, 1000, and 2500 mg/30 ml and contained full-spectrum CBD and trace THC. These drops were started with dosages that were calculated according to the patient’s body weight, with one sublingual drop twice a day and one drop every three days. The average daily CBD-enriched cannabis dose was 0.7 mg/kg (0.3–2 mg/kg). No patient was given a daily maintenance dose of CBD higher than 40 mg/day. The average duration of treatment was 6.5 months (3–28 months).

## Results and outcomes

The outcomes were evaluated before and after treatment based on clinical interviews. At each follow-up visit, parents were asked to assess the overall effectiveness of CBD-enriched cannabis treatment. According to the parents’ reports, no change in daily life activity was reported in 6 (19.35%) patients. The main improvements of the treatment were as follows: a decrease in behavioral problems was reported in 10 patients (32.2%), an increase in expressive language was reported in 7 patients (22.5%), improved cognition was reported in 4 patients (12.9%), an increase in social interaction was reported in 3 patients (9.6%), and a decrease in stereotypes was reported in 1 patient (3.2%). The parents reported improvement in cognition in patients who adhered to CBD-enriched cannabis treatment for over two years. The antipsychotic drug could be stopped only in one patient who showed mild ASD symptoms. No change could be made in other drug use and doses.

### Discontinuation and side effects

A 13-year-old male patient with severe autism had generalized seizures after using 5 mg sublingual CBD, and the drug was discontinued because of this side effect. The epileptic seizures persisted despite the discontinuation of the treatment. Interictal sleep EEG showed symmetrical bilateral frontotemporal sharp-slow wave complexes. The patient was regularly treated with valproic acid and remained seizure-free after starting this antiepileptic drug. CBD-enriched cannabis was also discontinued in a nine-year-old male patient with severe autism after two weeks because of a significant increase in stereotypes. No change in laboratory values related to CBD-enriched cannabis was found in any patient.

Restlessness was the only reported side effect in 7 (22%) out of 31 patients who continued treatment for at least three months, and the CBD-enriched cannabis dose was reduced in these patients. As the amount was reduced, restlessness decreased.

### A review of other studies

The popularity of CBD-enriched cannabis for the treatment of autism is increasing. Scoping reviews were done to achieve a broad and thorough examination of the literature in this area. Aran et al*.* (2019) were the first to retrospectively assess CBD-enriched cannabis effects on 60 children with ASD and severe behavioral problems using an open-label cohort study. The mean age was 11.8 ± 3.5 years; 82% of patients used psychiatric medications; 77% of patients had low cognitive function; and 23.3% of patients had epilepsy. All the children received CBD and THC in a 20:1 ratio. The mean total daily dose was 3.8 ± 2.6 mg/kg/day CBD and 0.29 ± 0.22 mg/kg/day THC for children who received three daily doses (*n*=44) and 1.8 ± 1.6 mg/kg/day CBD and 0.22 ± 0.14 mg/kg/day THC for children who received two daily doses (*n*=16). The doses were titrated over 2–4 weeks. The mean follow-up period was 10.9 ± 2.3 months. Efficacy was assessed using the Caregiver Global Impression of Change (CaGI) scale. Considerable improvement in behavioral problems was noticed in 61% of patients. Improvement in anxiety and communication problems was seen in 39 and 47%, respectively. Based on these promising results, Aren et al. launched a new placebo-controlled crossover trial. This study is ongoing, and new outcomes will be addressed in future publications (Aran et al. 2019).

Another study was conducted to evaluate the efficacy and safety of CBD-enriched cannabis effects on autism. This prospective, open-label study was carried out by Lihi Bar-Lev Schleider et al. and included 188 patients. The mean age was 12.9 ± 7 years. A total of 14.4% of patients had epilepsy. Most patients used preparations with 30% CBD and 1.5% THC, and the average concentrations of CBD and THC were 79.5 ± 61.5 mg and 4.0 ± 3.0 mg, respectively. After one month of treatment, 179 patients adhered to the treatment, and only 119 patients could be evaluated. Significant improvement was reported in 48.7% of patients, moderate improvement was reported in 31.1% of patients, and no change was reported in 14.3% of patients. Side effects were reported in 5.9% of patients. After 6 months of treatment, 155 patients continued treatment with CBD. Of the latter group, 93 patients responded to the questionnaire, 30.1% reported significant improvement, 53.7% reported moderate improvement, 6.4% reported slight amelioration, and 8.6% of the patients reported no change. Quality of life, mood, and ability to perform daily living activities were evaluated before the treatment and at 6 months. A total of 31.3% of the patients reported good quality of life before treatment. After 6 months, this percentage increased up to 66.8% (Lihi Bar-Lev Schleider et al. 2019).

Paulo Fleury et al. (2019) conducted a prospective, observational, and open-label study with a cohort of 18 autistic patients who received CBD-enriched cannabis (with a CBD-to-THC ratio of 75/1). The average dose of CBD was 4.55 mg/kg/day (a minimum of 3.75 mg and a maximum of 6.45 mg/kg/day). The average THC dose was 0.06 mg/kg/day (a minimum of 0.05 and a maximum of 0.09 mg/kg/day). The mean age was ten years. Fifteen patients adhered to the treatment (10 nonepileptic and five epileptic), and only one patient showed a lack of improvement in autistic behaviors. The most significant improvements were reported for seizures, attention-deficit/hyperactivity disorder, sleep disorders, communication, and social interaction (Paulo Fleury et al. 2019). Barchel et al. (2019) performed an open-label study on 53 autistic children. The median age was 11 (4–22) years; these patients received CBD at a concentration of 30% and a 1:20 ratio of CBD to THC. The median THC interquartile range (IQR) daily dose was 7 (4–11) mg, and the median CBD (IQR) daily dose was 90 (45–143) mg. The median duration of treatment was 66 days (30–588). Self-injury and rage attacks improved by 67.6% and worsened by 8.8%, respectively. Improvement in hyperactivity symptoms was reported in 68.4% of patients, 28.9% reported no change, and 2.6% reported worsening symptoms. Sleep problems improved by 71.4% and worsened by 4.7%. There was an improvement in anxiety in 47.1% and worsening in 23.5% of patients (Barchel et al. 2019). Mojdeh Mostafavi et al. (2020) reported positive effects of cannabis in ASD, especially in aggressive and self-injurious behaviors (Mostafavi and Gaitanis 2020). McVige et al. (2020) carried out an important retrospective and open-label study on 20 patients with ASD (6 with epilepsy and 14 with pain). These patients were on cannabis treatment. The study reported very significant positive outcomes. The Autism/Caregiver Global Impression of Change (ACGIC) scale revealed improvements in sleep, mood, and aggression toward the self or others; there were also improvements in patient communication abilities and attention/concentration (McVige et al. 2020).

According to Aren et al.’s study, adverse events such as hypervigilance aggravated sleep disturbances in 14% of patients. This side effect was resolved by omitting or adjusting the evening doses. Irritability in 9% and loss of appetite in 9% were seen. A thirteen-year-old girl received 6.5 mg/kg/day CBD and no other medications; when she gradually increased the THC dose up to 0.72 mg/kg/day, she developed sudden behavioral changes such as unusual vocalization and refusal to sleep and eat for two days. The symptoms resolved when she stopped CBD and THC and received antipsychotic treatment (ziprasidone). After cannabis treatment, psychiatric medications were regulated in most patients; 33% received fewer or lower doses, 24% stopped taking medications, and 8% received more medication or higher doses (Aran et al. 2019). Lihi Bar-Lev Schleider et al. reported mild side effects such as restlessness, sleepiness, dry mouth, and digestion problems (Lihi Bar-Lev Schleider et al. 2019). Paulo Fleury et al*.* reported that three patients stopped using CBD-enriched cannabis in a period shorter than one month due to side effects (autistic behaviors had worsened in two patients, which might happen due to the unsupervised and sudden cessation of the antipsychotics; one patient had insomnia, irritability, increased heart rate, and worsening of psych-behavioral crises that might be due to the interaction of cannabis with previous prescribed antipsychotic drugs). Mild and transient adverse effects such as sleepiness, moderate irritability, diarrhea, increased appetite, conjunctival hyperemia, and increased body temperature were also reported (Paulo Fleury et al. 2019).

## Discussion

In the updated review, preliminary evidence announcing that cannabinoids (compounds with different ratios of CBD and THC) could exert beneficial effects on some ASD-associated symptoms, such as behavioral problems, hyperactivity, and sleep disorders, with a lower number of metabolic and neurological side effects than approved medications. Importantly, treatment with cannabinoids permits a reduction in the number of prescribed drugs and significantly reduces the frequency of seizures in participants with comorbid epilepsy. In this paper, we aimed to make some critical points related to the main findings and mechanisms of action of cannabinoids, such as a decrease in behavioral problems, an increase in the expressive language, an improvement in cognition, and an increase in social interaction when patients used CBD-enriched cannabis at a dose of 0.7 mg/kg (0.3–2 mg/kg), which is lower than the doses reported in other studies. Furthermore, these results are consistent with other studies that suggest that supplementing ASD patients with CBD-enriched cannabis could improve behavioral problems. A dose of 3.8 ± 2.6 mg/kg/day CBD was used in Aren et al.’s study and yielded improvements in anxiety and communication problems. According to Paulo Fleury et al., the average dose of CBD was 4,55 mg/kg/day, and the results showed that only one patient reported no improvement in autistic behaviors. The most significant improvements were reported for seizures, attention-deficit/hyperactivity disorder, sleep disorders, communication, and social interaction. In addition, improvements in expressive language were seen. CBD-enriched cannabis might help children with ASD via several possible mechanisms, including its anxiolytic and antipsychotic properties and its impact on the endocannabinoid system (ECS) and oxytocin (Dos Santos et al. 2019; McVige et al. 2020; Premolia et al. 2019). According to our results, we recommend using lower doses of CBD-enriched cannabis.

CBD use is not devoid of health risks; known risks include liver damage, adverse effects on the male reproductive system, potential drug interactions that may be associated with adverse events or diminished efficacy of approved therapies, and additional unknown health risks. However, the pharmacology of CBD has not been well studied; thus, little is known about both the potential therapeutic benefits and the hazards of short- or long-term use (Leas et al. 2020). According to our study, restlessness was the only mild side effect seen in some patients which was resolved on making some doses adjustments. In addition, generalized seizures after starting CBD-enriched cannabis. And these seizures re-occurred even several months after cessation of CBD treatment, and abnormal EEG results were seen. Therefore, this study cannot make causal inferences on the relation between CBD-enriched cannabis and seizures. Not all patients benefit equally from the use of CBD. The reason why some patients experienced benefits while others experienced side effects could be due to candidate genes that may influence the acute effects of cannabis. Genes posited to have specific influences on cannabis include CNR1, CB2, FAAH, MGL, TRPV1, and GRP55. When some patients have a mutation in these receptors, different results could be seen when cannabis was used (Agrawal and Lynskey 2009). Other studies also reported reversible and some mild side effects, none of which were life-threatening. Most of the side effects were overcome by adjusting the doses. Furthermore, the use of recreational cannabis in adolescents is associated with several risks, including decreased motivation, addiction, mild cognitive decline, and schizophrenia. However, these complications are all attributed to THC. Our study drug was full-spectrum CBD and trace THC. Nevertheless, systematic evaluation of safety data of CBD use in children is still lacking. Future research is recommended that examines the clinical impact of CBD-enriched cannabis. Additionally, rarer side effects were seen in our patients compared to other studies, which could be due to using lower doses of CBD and trace THC (a brief overview of all these studies is given in Tables 1 and 2).

**Table 1:** Characteristics of the included studies/efficacy of cannabis-related drugs in ASD

Study characteristics		Characteristics of patients		Treatment characteristics			
Authors/year	Study design	No. of ASD participants	Mean age years	Active treatment doses/mg/day	Daily dosage mg/kg/day	Mean follow-up months	Benefits No. of patients or (%)
Serap-Ekici et al. (2021), this study Retrospective	Retrospective	33	7.7 ± 5.5	Synthetic CBD	0.7 (0.3–2) max 40 mg/day	6.5	10 (32.2%) patients reported a decrease in behavior problems 7 (22.5%) patients reported an increase in expressive language 4 (12.9%) patients reported an improvement in cognitive functions 3 (9.6%) patients reported an increase in social interaction 1 (3.2%) patient reported a decrease in stereotypes 6 (19.3%) patients reported no change
Aran et al. (2019)	Retrospective Cohort study Open-label	60	11.8 (5–17.5)	Cannabinoid oil A solution at a 20:1 ratio of CBD and THC	Sublingual assumption 2 or 3 times/daily with CBD doses stated at 1 mg/kg/day and titrate up to 10 mg/kg/d stated at 1 mg/kg/day stated at 1 mg/kg/day	10.9	61% of patients reported an improvement in behavior problems 39% of patients reported an improvement in anxiety 47% of patients reported an improvement in communication 33% of patients received fewer medications or lower doses 24% of patients stopped psychotic medications 8% of patients received more medications
Lihi Bar-Lev Schleider et al. (2019)	Prospective	188	12.9	Most patients consumed oil with 30% CBD and 1.5% THC. Insomnia was treated with an the evening dose of 3% THC oil	On average 79.5 ± 61.5 mg CBD and 4.0 ± 3.0 mg THC, three times a day. Average additional 5.0 ± 4.5 mg THC daily for insomnia	6	Quality of life: 66.8% of patients reported an improvement Mood: 63.5% of patients reported an improvement Adaptive abilities: 42.9% of patients reported an improvement Concentration: 14% of patients reported an improvement
Paulo Fleury et al. (2019)	Prospective Cohort study	18 (15 analyzed)	10.9 (6–17)	Sativa extract containing 75:1 CBD:THC ratio	CBD: mean 175 mg/day (100–350); THC: 2.33 mg/day (1.33–2.33).	12.4	30% of patients reported an improvement in ADHD 20% of patients reported improvements in behavior disorder 25% of patients reported improvements in communication, social, and interaction deficit 20% reported improvements in cognitive function 40% reported improvements in sleep disorder
Barchel et al. (2019)	Prospective Cohort study	53	11 (4–22)	Cannabinoid oil solution at a concentration of 30% and 20:1 ratio of CBD and THC	CBD: 16 mg/kg (maximal daily, dose 600 mg) THC: 0.8 mg/kg (maximal daily, a dose of 40 mg).	66 days (30–588)	Hyperactivity: Improvement: 68.4% No change: 28.9% Worsening: 2.6% - Self-injury: Improvement: 67.6% No change: 25% Worsening: 8.8% - Sleep problems: Improvement: 71.4% No change: 23.8% Worsening: 4.7% - Anxiety: Improvement: 47.1% No change: 29.4% Worsening: 23.5% Overall: improvement: 74.5%; no change: 21.6%; Worsening: 3.9%
Mostafavi and Gaitanis (2020)	Retrospective Preliminary	32	Not reported	Medical marijuana or hemp-based products	None	None	91% improvement in seizures 60% improvement in aggression
Mc Vige et al. (2020)	Retrospective	20	Not reported	Medical cannabis	None	Improvement in the degree of overall pain	Improvement in seizure frequency and severity Improvement in sleep, mood, aggression toward self and/or others, communication abilities and 50% of patients discontinued or reduced the use of other medication

**Table 2:** Characteristics of participants of patients, comorbidities, and reported side effects

Author/year	Characteristic of/comorbidity of patients	Characteristic of/comorbidity of patients	Discontinuations/reason
Serap-Ekici et al. (2021), this study	18 (54.6%) heavy autism 15 (45.4%) mild autism 3 (9%) epilepsy 7 (21%) abnormal EEG	Restlessness in 7 (22,5%) resolved after resolved after adjusting the dose, seizures increase in stereotypes	2 (6%) seizures *n*=1 Increase in stereotypes *n*= 1
Aran et al. (2019)	77% low intellectual disability 14 (23.3%) epilepsy	Any adverse event (51%), sleep disturbances (14%), restlessness (9%), nervousness (9%), loss of appetite (9%), gastrointestinal symptoms (7%), unexplained laugh (7%), mood changes (5%), fatigue (5%), nocturnal enuresis (3.5%), tremor (3.5%), sleepiness (2%), anxiety (2%), confusion (2%), psychotic event (2%)	1 (1.6%) Severe psychotic events
Lihi Bar-Lev Schleider et al. (2019)	27 (14.4%) epilepsy Antipsychotics (56.9%),	Restlessness (6.6%), sleepiness (3.2%), psychoactive effect (3.2%), increased appetite (3.2%), digestion problems (3.2%), dry mouth (2.2%), lack of appetite (2.2%)	23 (12.2%) no therapeutic effect and side effects, some patients intended to return to treatment again
Paulo Fleury et al. (2019)	5 (27.7%) epilepsy (46.7%) antipsychotics	Sleepiness, moderate irritability (*n* = 3); diarrhea, increased appetite, conjunctival hyperemia, and increased body temperature (*n* = 1) Nocturia (*n* = 2), which in one case, the heart rate increase, psycho-behavioral problems	3 out of 18 (16.7%) psycho-behavioral effects and increased heart rate
Barchel et al. (2019)	Atypical antipsychotics (58.4%),	Somnolence (22.6%), appetite decrease (11.3%), appetite increase (7.5%), insomnia (3.7%), sense abnormality response (to temperature) (3.7%), diarrhea (3.7%), hair loss (1.8%), nausea (1.8%), confusion (1.8%)	5 (9.4%)
Mostafavi and Gaitanis (2020)	(63%) developmental regression	Increases in OCD, repetitive behaviors, insomnia, mania (3 on Hemp oil, one on medical marijuana)	None
	69%) epilepsy (63%) developmental delay	Three patients reported mild adverse events (unspecified).	None
McVige et al. (2020)			

These preclinical data and the current study results render further exploration of this treatment avenue in controlled studies. Until such evidence is available, physicians should be cautious when using medical cannabis to treat children with ASD since initial reports of promising treatment in children with ASD are often found.

### Limitations of the study

The absence of the control study group, the use of various strains of CBD-enriched cannabis extracts, different durations of treatment and dosages, and depending on the reports of the parents instead of standard assessment scales are considered to be the main limitations of the study. The clinical assessments were done with knowledge of the patients’ treatment (it was an open-label case series, not a blinded clinical trial.

## Conclusion

Using lower doses of CBD and trace THC seems to be promising in the management of behavioral problems associated with autism. In addition, this treatment could be effective in managing core symptoms and cognitive functions. No significant side effects were seen at the low doses of CBD-enriched cannabis when compared to other studies.

## Data Availability

The datasets used and analyzed in this review article are available from the corresponding author upon reasonable request.

## Abbreviations

ASD: Autism spectrum disorder
ECS: Endocannabinoid system
CBD: Cannabidiol
THC: Tetrahydrocannabinol
FDA: Food and Drug Administration
eCBs: Endocannabinoids
GPR: G protein-coupled receptor
CB: Cannabinoid receptor
TRPV1: Transient receptor potential cation channel subfamily V member 1
FAAH: Fatty acid amide hydrolase
PPAR-γ: Peroxisome proliferator-activated receptor-gamma
GPR55: G Protein-coupled receptor 55
ROS: Reactive oxygen species
OEA: Oleoylethanolamide
MC4R: Melanocortin 4 receptor
NAc: Nucleus accumbens
ACTH: Adrenocorticotropic hormone
CaGI: Caregiver Global Impression of Change
EEG: Electroenceography
